# Identification of *LiMYC* and *LiTPS* Gene Families Involved in MeJA-Induced Terpene Accumulation in *Lagerstroemia indica* ‘Whit III’

**DOI:** 10.3390/plants15111600

**Published:** 2026-05-22

**Authors:** Jingyun Wang, Hao Dou, Ziwei Yue, Yan Xu, Ming Cai

**Affiliations:** Beijing Key Laboratory of Ornamental Plants Germplasm Innovation & Molecular Breeding, National Engineering Research Center for Floriculture, School of Landscape Architecture, Beijing Forestry University, Beijing 100083, China; jingyunwong@bjfu.edu.cn (J.W.); douhao@bjfu.edu.cn (H.D.); yueziwei@bjfu.edu.cn (Z.Y.); xuyan@bjfu.edu.cn (Y.X.)

**Keywords:** *Lagerstroemia indica*, methyl jasmonate, terpene synthesis, MYC transcription factor, floral volatile

## Abstract

Methyl jasmonate (MeJA) is a key regulator of plant defense and abiotic stress responses, while terpenoids are important secondary metabolites. However, the effects of MeJA on floral volatiles in *Lagerstroemia indica* and the underlying mechanisms remain unclear. In *L. indica* ‘Whit III’, MeJA treatment rapidly increased the emission of monoterpenes (e.g., citronellol) and sesquiterpenes (e.g., *trans*-farnesol) and advanced the peak emission time. We identified 34 *LiTPS* and 22 *LiMYC* genes in the genome, with promoter regions enriched in JA-responsive *cis*-elements. Expression analysis showed that *LiMYC* genes encoding putative JA repressors were transiently upregulated, whereas *LiTPS* genes located in a chromosome 11 cluster and *LiTPS13* (7.33-fold induction) were strongly activated. These results suggest that MeJA may promote an early scent production through the coordinated activation of specific *LiMYC* and *LiTPS* gene sets, pointing to a potential mechanism by which jasmonate signaling modulates floral volatile emission.

## 1. Introduction

As sessile organisms, plants have evolved sophisticated chemical defense systems to navigate complex and fluctuating environments [1]. Secondary metabolites (SMs) play a pivotal role in this arsenal, serving not only as the first line of defense against biotic stresses (e.g., herbivory, pathogens) and abiotic stresses (e.g., heat, drought), but also as a chemical language for plant–environment communication [2]. Among these, volatile organic compounds (VOCs), particularly terpenoids, function in both direct defense (e.g., deterring herbivores) and indirect defense (e.g., recruiting natural enemies of pests) [3,4]. Elucidating how plants reprogram terpenoid biosynthesis to produce defensive volatiles in response to environmental cues remains a central question in plant physiology and chemical ecology.

Plant stress responses are orchestrated by hormonal signaling networks [5], in which jasmonic acid (JA) acts as a central regulator of defense [6]. Upon wounding or herbivore attack, endogenous JA levels surge rapidly, triggering the canonical “COI1-JAZ-MYC” signaling module [7,8,9,10]. In this pathway, the bioactive conjugate JA-Ile is perceived by the receptor complex, leading to the ubiquitination and degradation of JAZ repressor proteins. This releases downstream basic helix–loop–helix (bHLH) transcription factors to activate defense gene expression [11].

The bHLH family, one of the largest transcription factor families in eukaryotes, plays key roles in plant development and stress adaptation. Within this family, the Myelocytomatosis (*MYC*) subfamily conserved HLH and bHLH-MYCN domains [12]. In *Arabidopsis thaliana*, *MYC* genes are functionally classified into three main clades: the activator clade (*AtMYC2*, *AtMYC3*, and *AtMYC4*), which governs JA-mediated defense responses [13]; the repressor (*AtJAM1*-*AtJAM4*), which negatively regulates JA signaling to maintain metabolic homeostasis [14]; and *AtMYC1*, which primarily functions in epidermal development [15]. Upon release, free MYC proteins translocate to the nucleus, G-box (CACGTG) in target promoters, activating transcription of defense-related genes, including terpene synthases (*TPS*) [16,17].

The MYC–TPS regulatory module is functionally conserved and has been experimentally validated in several non-model species. For instance, MYC2 has been shown to bind *TPS* promoters and regulate their expression, thereby influencing volatile terpenoid accumulation in crops and ornamentals such as rice [18], *Osmanthus fragrans* [19], *Hedychium coronarium* [20], and *Chrysanthemum* [8]. However, despite the functional conservation, the composition and dynamic regulation of this network remain unexplored in *L*. *indica*, a species of ornamental [21] and medicinal [22] value.

Specifically, for *L. indica* ‘Whit III’, which emits only a faint floral scent under natural conditions, it remains unclear whether its biosynthetic capacity for defensive volatiles is present but not actively engaged. Exogenous methyl jasmonate (MeJA) is known to activate plant defense responses and can serve as a tool for studying the regulation of secondary metabolism. However, whether MeJA can shift the balance between activator and repressor *MYC* members, and whether this leads to activation of specific LiMYC transcription factors that induce *LiTPS* gene expression and terpenoid accumulation, remain to be addressed.

Although the JA-TPS pathway has been characterized in other species, it remains unclear whether *L. indica*, especially the low-scent *L. indica* ‘Whit III’, retains the ability to produce defensive terpenes in response to JA signaling, and if so, how MeJA may activate this underlying MYC–TPS regulatory relationship. To address these questions, this study combined volatile metabolite profiling with genome-wide identification and expression analysis of *LiTPS* and *LiMYC* genes in *L. indica* ‘Whit III’ aiming to investigate the mechanisms by which MeJA induced defensive volatile synthesis.

Our specific objectives were to: (1) characterize the temporal changes in floral volatile emissions in *L. indica* ‘Whit III’ under control group and following MeJA treatment, and identify terpenoids responsive to treatment; (2) identify the *LiTPS* and *LiMYC* gene families at the genome-wide level, and use phylogenetic analysis, domain characterization, and promoter *cis*-motif prediction to identify candidate genes potentially putatively involved in stress responses; and (3) explore potential regulatory relationships linking MeJA signaling, LiMYC transcription factors, and LiTPS synthases. This study provides a foundation for understanding the molecular basis of floral scent regulation in *L. indica* and offers candidate genetic resources for future functional studies on stress resilience and the utilization of high-value compounds.

## 2. Results

### 2.1. Effects of Exogenous MeJA on Floral Volatile Composition

#### 2.1.1. Temporal Changes in Total Volatile and Terpenoid Emissions

**Figure 1:**
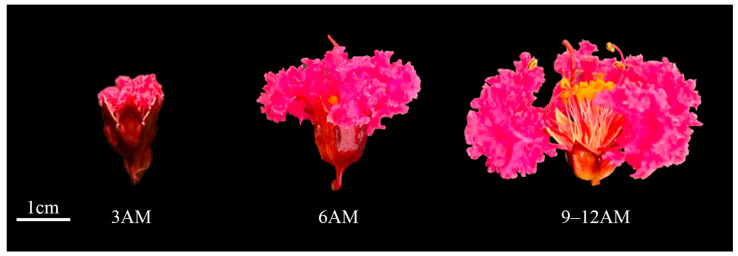
Flower morphology of *L. indica* ‘Whit III’ at full anthesis.

**Figure 2:**
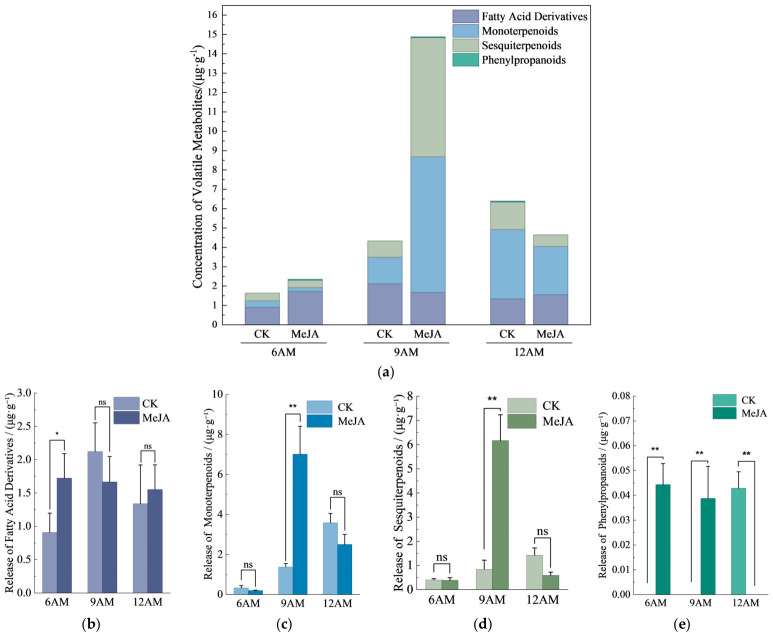
Temporal dynamics of volatile metabolite profiles in *L. indica* ‘Whit III’ flowers under exogenous 0.25% MeJA treatment. (**a**) Total concentration and chemical class composition of volatiles at 6:00, 9:00, and 12:00. Release patterns of specific compound classes: fatty acid derivatives (**b**), sesquiterpenoids; (**c**), monoterpenoids; (**d**), and phenylpropanoids (**e**). Data are presented as mean ± SD *n* = 3. Asterisks indicate significant differences between CK and 0.25% MeJA treatments at each time point (* *p* < 0.05, ** *p* < 0.01; ns, not significant).

Previous studies indicated that volatile emission in *Lagerstroemia* species typically peaks between 06:00 and 09:00 [23,24]. However, in *L. indica* ‘Whit III’, the maximum accumulation in the control group (CK) occurs later, between 09:00 and 12:00 during full anthesis (Figure 1). In CK flowers, volatile accumulation showed a diurnal rhythm and a shift in chemical composition. As shown in Figure 2a, at 06:00 (early anthesis), total volatile levels were low, dominated by fatty acid derivatives (55% of total); monoterpene levels were barely detected. As flowers opened, volatile accumulation increased, reaching a peak total concentration of 6.39 μg·g^−1^ at 12:00. At this peak, monoterpenes accumulated to 3.58 μg·g^−1^, replacing fatty acid derivatives as the dominant class (56%), indicating a shift toward a more monoterpene-dominated profile.

Exogenous MeJA treatment significantly altered this natural accumulation pattern. MeJA residues were detected in treated floral tissues from 06:00 to 09:00, confirming exposure to MeJA. MeJA not only increased the total amount of volatile emission but also advanced the timing of the emission peak. Unlike the CK group, which peaked at 12:00, the MeJA-treated group reached its maximum total concentration (14.88 μg·g^−1^) at 09:00. This value represented a 3.43-fold increase compared to the CK group at the same time point (4.34 μg·g^−1^), indicating that MeJA induced more rapid accumulation.

MeJA also altered the volatile composition toward a higher proportion of terpenoids. As shown in Figure 2c,d, both monoterpene and sesquiterpene levels were elevated following MeJA treatment. At 09:00, total monoterpene content in the MeJA group reached 7.01 μg·g^−1^, compared to 1.37 μg·g^−1^ in the CK group, representing a 5.12-fold increase. Sesquiterpenes reached 6.16 μg·g^−1^, 7.33-fold higher than the CK group (0.84 μg·g^−1^). Both terpenoid classes accounted for a larger share of the total volatile profile at this time point. In contrast, the relative proportion of fatty acid derivatives, which dominated the CK profile, was significantly reduced by MeJA treatment. These results indicate that MeJA treatment led to a greater increase in terpenoid emission compared to other volatiles, resulting in a terpenoid-dominated volatile profile at an earlier stage (09:00). The detailed volatile profiling data are provided in Supplementary Table S1.

#### 2.1.2. Changes in Key Individual Compounds and Overall Volatile Profiles

**Figure 3:**
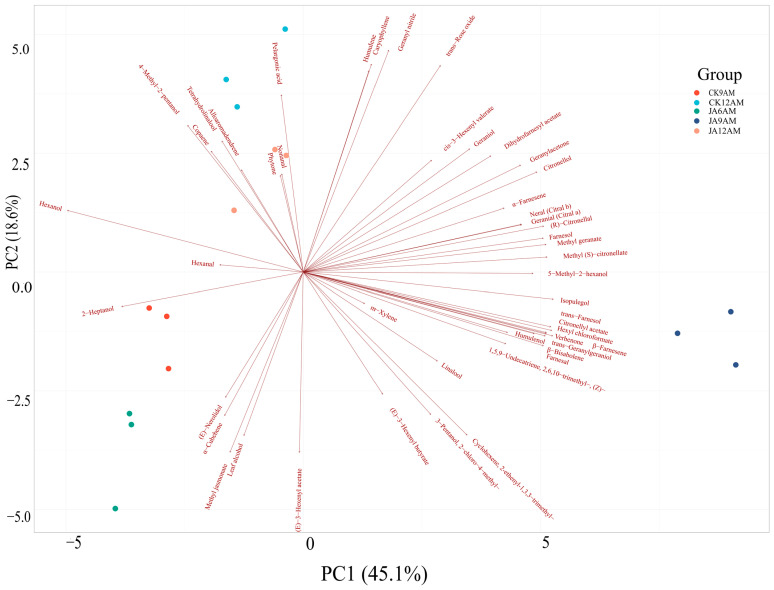
Principal component analysis (PCA) biplot of floral volatiles in *L. indica* ‘Whit III’ under CK and 0.25% MeJA treatments, highlighting key compound contributions.

To further identify individual compounds responsive to MeJA, we quantified the major volatiles constituents. Among more than 30 detected compounds, several showed increased levels following the MeJA treatment (Figure 3). The monoterpenes, citronellol and geraniol, increased substantially in the treated group. At 09:00, citronellol content reached 2.46 μg·g^−1^ in the MeJA group, compared to 0.45 μg·g^−1^ in the CK group (5.47-fold). Geraniol levels increased from 0.06 μg·g^−1^ (CK) to 0.26 μg·g^−1^ (MeJA), a 4.33-fold increase.

Among sesquiterpenes, *trans*-farnesol reached 4.47 μg·g^−1^ at 09:00 in the MeJA group, compared to 0.48 μg·g^−1^ in the CK group (9.31-fold increase). Methyl geranate increased from 0.63 μg·g^−1^ (CK) to 2.90 μg·g^−1^ (MeJA), a 4.60-fold increase. These results indicate that MeJA was associated with increased accumulation of these terpenoids.

**Figure 4:**
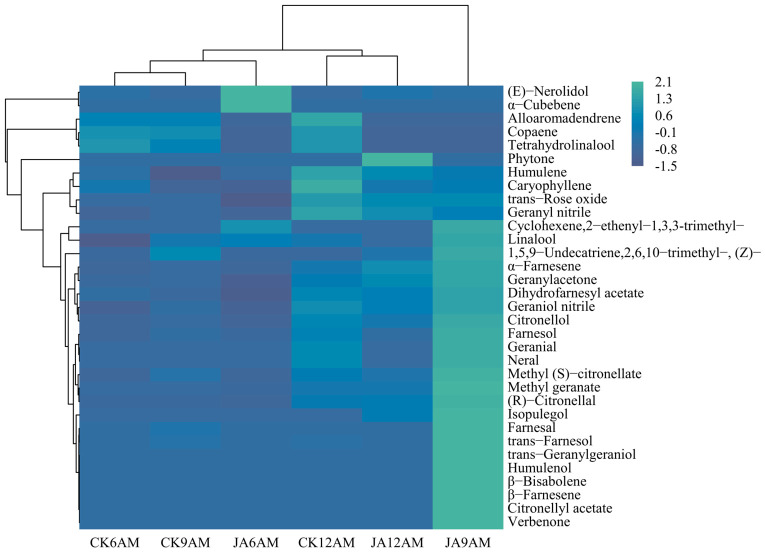
Heatmap showing spatiotemporal changes in floral volatile profiles of *L. indica* ‘Whit III’ induced by 0.25% MeJA treatment.

To examine the overall variation among samples, we performed principal component analysis (PCA) and hierarchical clustering. In the PCA score plot (Figure 3), CK and MeJA samples were separated along PC1, with the first two principal components accounting for 63.7% of the total variance. The 09:00 MeJA samples were distinctly separated from all CK time points. The analysis of factor loadings indicated that *trans*-farnesol, citronellol, and geraniol were the main contributors to this separation. The heatmap (Figure 4) further showed that the upregulation of terpenoids was consistent across biological replicates.

### 2.2. Genomic Identification and Evolutionary Characteristics of the TPS and MYC Gene Families in L. indica

#### 2.2.1. Chromosomal Localization and Gene Cluster Distribution

**Figure 5:**
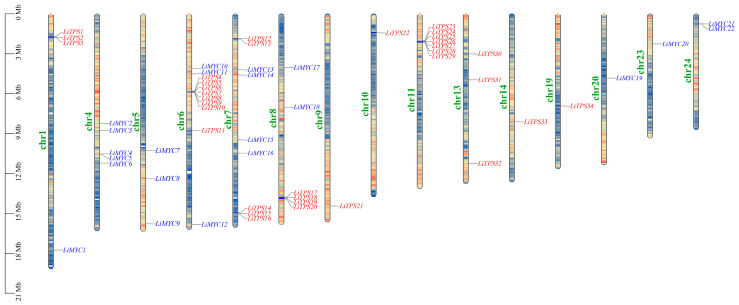
Chromosomal distribution of *LiTPS* and *LiMYC* genes across the 15 chromosomes of *L. indica.* Red indicates *LiTPS* genes, and blue indicates *LiMYC* genes.

To identify genes potentially involved in floral volatile synthesis in *L. indica* ‘Whit III’, we performed a genome-wide identification of terpene synthase (*LiTPS*) and MYC transcription factor (*LiMYC*) genes. A total of 34 *LiTPS* genes and 22 *LiMYC* genes were identified. Both gene families exhibited a non-uniform distribution across the 15 chromosomes of *L. indica*. *LiTPS* genes were concentrated on chr6 (eight members) and chr11 (seven members), forming tandem clusters such as the *LiTPS4–LiTPS10* cluster on chr6 and the *LiTPS23–LiTPS29* cluster on chr11. Smaller tandem arrangements were also observed on chr1, chr7, and chr8. In contrast, chr2, chr3, and chr12 contained no *LiTPS* members. Conversely, the *LiMYC* family was mainly distributed on chr4 (five members) and chr7 (four members) and did not form distinct tandem clusters (Figure 5).

Physicochemical properties of the proteins were predicted using ExPASy tools (Table S2). The CDS lengths of *LiTPS* genes ranged from 1437 bp (*LiTPS29*) to 2589 bp (*LiTPS34*), with the exception of *LiTPS33* (5727 bp). Their predicted molecular weights ranged from 55.58 kDa (LiTPS29) to 97.44 kDa, except for LiTPS33. Predicted isoelectric points ranged from 5.03 (LiTPS26) to 6.98 (LiTPS25). All LiTPS proteins had negative grand average of hydropathicity (GRAVY) values, suggesting they are predicted to be hydrophilic. Subcellular localization prediction indicated that the majority of LiTPS proteins were predicted to localize in chloroplasts (61.76%) and cytoplasm (20.59%), with a minor proportion in the nucleus, peroxisomes, and plasma membrane.

Although co-localization of both families occurs on chr1, chr6, chr7, and chr8, their physical positions does not show extensive overlap, suggesting largely independent chromosomal distributions. The tandem clustering of the *LiTPS* genes may indicate that tandem duplication has contributed to the expansion of this family. Such gene dosage effect provides a genetic basis for the terpenoid diversity observed in the volatile profiles, through functional validation is required to establish a direct link.

#### 2.2.2. Phylogenetic Relationships and Conserved Domain Analysis

**Figure 6:**
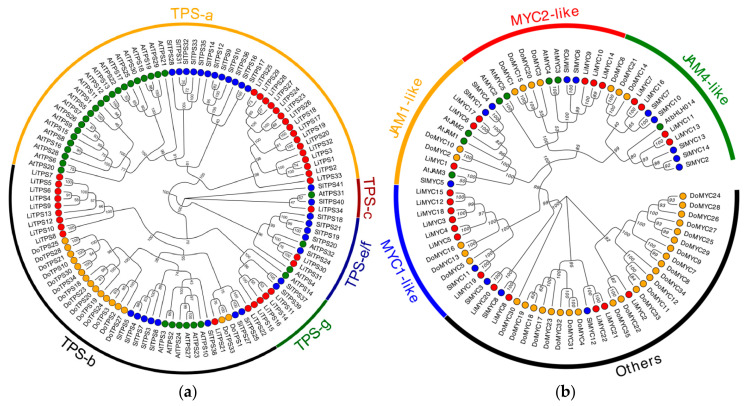
Phylogenetic relationships of (**a**) TPS and (**b**) MYC proteins from *L. indica* and related species. Bootstrap values (>70%) are shown at nodes. In (**a**), the TPS proteins are classified into five subfamilies: TPS-a, TPS-b, TPS-c, TPS-e/f, and TPS-g. Species abbreviations: At, *Arabidopsis thaliana*; Si, *Solanum lycopersicum*; Do, *Dendrobium officinale*. In (**b**), the MYC proteins are grouped according to their putative functions: MYC2-like (JA activators), JAM-like (JA repressors), MYC1-like (trichome development), and other members not directly associated with JA signaling.

Phylogenetic trees for the *TPS* and *MYC* families were constructed from four representative species using the Neighbor-Joining (NJ) method. Most internal branches received bootstrap support above 70%, separating the sequences into distinct clades (Figure 6).

The *LiMYC* members were dispersed across various clades rather than forming a single, species-specific monophyletic group. Most *LiMYC* proteins clustered tightly with their orthologs from *D. officinale* (*DoMYC*) and tomato (*SlMYC*), while others grouped with *A. thaliana* (*AtMYC*) sequences. This interspersed topology suggests an ancient origin within dicots and indicates a high degree of sequence conservation throughout evolution, implying that the core regulatory functions of these transcription factors have been maintained since before the divergence of these lineages.

In contrast, the *TPS* phylogenetic tree exhibited a more pronounced hierarchical structure, resolving into seven major evolutionary clades. Notably, *LiTPS* members within the same clade—particularly those originating from the chr11 tandem cluster—tended to form tight intra-species sub-clusters. This phylogenetic pattern aligns perfectly with the chromosomal localization data, providing strong independent validation that recent tandem duplication events are the primary driver behind the expansion and diversification of the *TPS* family in *L. indica*. The clustering of paralogs within species-specific branches further underscores the role of lineage-specific gene amplification in shaping the terpenoid biosynthetic capacity of *L. indica*.

**Figure 7:**
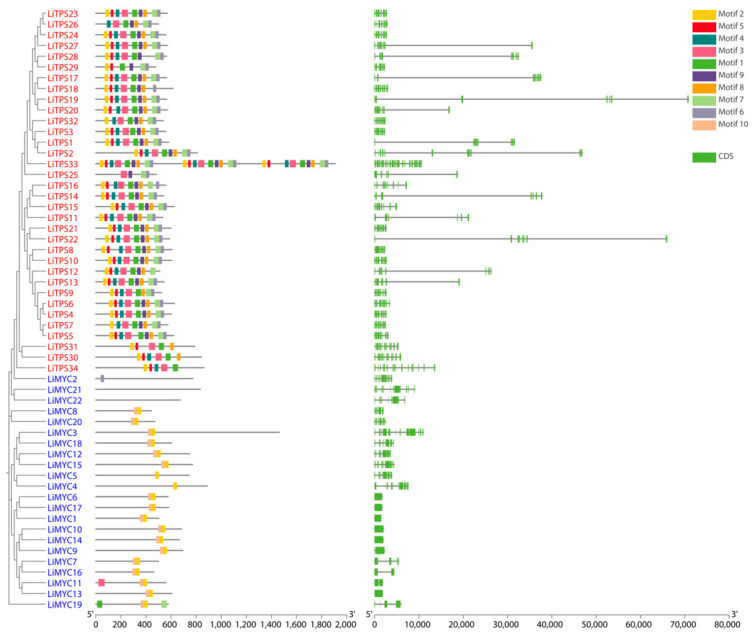
Conserved motif identification and gene structure analysis of *LiTPS* and *LiMYC* genes in *L. indica*. (**Left**): phylogenetic tree and distribution of conserved motifs (Motif 1–10) identified by MEME. Each colored box represents a specific motif. (**Right**): exon–intron structures of corresponding genes. Green boxes represent coding sequences (CDS), and gray lines represent introns. The scale bar indicates sequence length (bp). The 5′ and 3′ orientations are shown at the bottom. Red indicates *LiTPS* genes, and blue indicates *LiMYC* genes.

Further analysis of gene architecture and conserved motifs elucidated the critical correspondence between structural complexity and functional specialization (Figure 7).

The *LiTPS* family showed a split gene structure with variable gene length (from 2 to 80 kb), a high number of exons (5–20), and extensive intronic regions. The conserved motifs (Motif 1–10) were arranged in a similar across members; members within the same phylogenetic clade (e.g., *LiTPS23–LiTPS29*) share an identical motif composition. This structural uniformity similarity is consistent with a duplication–retention model, where duplicated genes are preserved without major structural divergence. *LiTPS33*, a putative sesquiterpene synthase belonging to the TPS-a subfamily, contains three tandemly repeated *TPS* domains, an unusual feature in plant *TPS* genes. Further characterization of this unusual domain architecture may provide insights into the functional diversification of the *TPS* family in *L. indica*.

In contrast, the *LiMYC* family displayed a compact genomic structure. Nearly half of the *LiMYC* members are single-exon genes, with total lengths generally under 20 kb. Furthermore, they contain only one to three conserved motifs, with Motif 2 predicted as the core functional domain. This compact organization may facilitate rapid transcriptional responses upon signal induction.

Collectively, these structure function suggest distinct evolutionary trajectories for the two families. The more complex exon–intron organization and motif diversity in *LiTPS* genes, together with the compact structure and fewer motifs in *LiMYC* genes, are consistent with their proposed roles in biosynthesis and transcriptional regulation, respectively. Whether these structural differences directly reflect functional specialization requires experimental investigation.

#### 2.2.3. Analysis of Cis-Regulatory Elements in Promoter Regions

**Figure 8:**
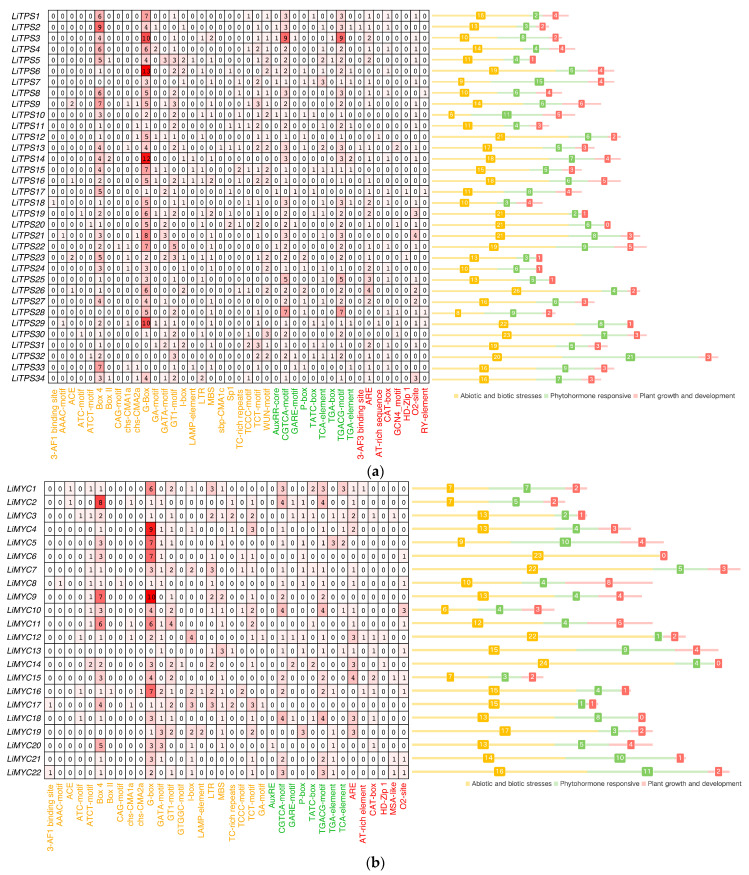
*Cis*-regulatory elements in the promoter regions of *LiTPS* (**a**) and *LiMYC* (**b**) genes. Left: heatmap of *cis*-acting element counts, with color intensity representing element abundance. Right: functional classification and positional distribution of elements. Yellow, green, and red blocks indicate elements related to stress responses, phytohormone responses, and plant growth/development, respectively.

To identify putative cis-regulatory elements that may contribute to MeJA-mediated regulation of *LiTPS* and *LiMYC* genes, we predicted motifs within the 2000 bp region upstream of the translation start site using the PlantCARE database (Figure 8).

Both families shared predicted binding sites for G-boxes, and MYC recognition motifs, suggesting they may be co-regulated by upstream signaling networks. In the *LiTPS* promoters, G-boxes (15.7%), MYB-related elements (21.1%), ABREs (20.5%), and MYC-binding sites (14.2%) were among the most represented motifs. Among phytohormone-responsive elements, the JA-associated CGTCA-motif and TGACG-motif each accounted for 20% of the predicted hormone-related motifs. Notably, the *LiTPS* family exhibited a higher proportion of predicted JA-responsive elements (CGTCA-motif and TGACG-motif) compared with the *LiMYC* family (14.8% vs. 11.0% of total predicted motifs).

Furthermore, specific *LiTPS* genes, including *LiTPS3*, *LiTPS25*, *LiTPS26*, *LiTPS29*, *LiTPS30* and members of tandem duplication clusters such as *LiTPS6*, contained relatively high numbers of these MeJA-associated motifs, indicating that these genes may be more responsive to JA signaling.

Since the presence of a motif alone does not guarantee functional relevance, we also examined the positional distribution of TATA boxes and key hormone-responsive elements in selected *LiTPS* and *LiMYC* promoters to assess whether these elements are located within regions that may support transcriptional regulation (Figure S1). These predicted promoter features are consistent with the enhanced terpenoid emission observed following MeJA treatment, although experimental validation is needed to establish functional relevance.

#### 2.2.4. Synteny Analysis and Protein Interaction Networks

**Figure 9:**
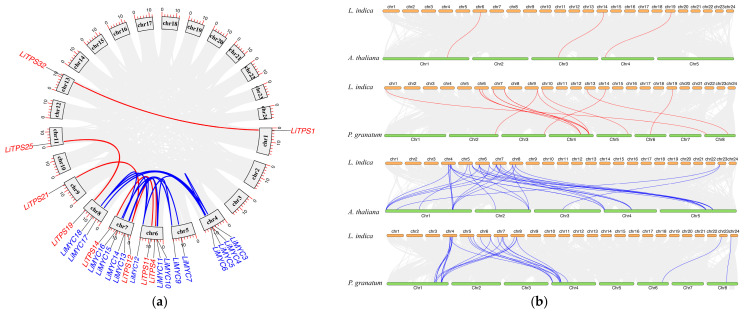
Synteny analysis of *TPS* and *MYC* genes (**a**) within *L. indica* and (**b**) between *L. indica* and selected plant species. The outer circle represents the 24 chromosomes (chr1–chr24). Red lines indicate collinear relationships between *LiTPS* family members, and blue lines indicate collinear relationships between *LiMYC* family members.

Intra-genomic synteny analysis identified 18 paralogous gene pairs within the *L. indica* genome, distributed mainly on chromosomes 4, 5, 6, 7, and 8 (Figure 9). The *LiMYC* family accounted for 13 pairs, while the *LiTPS* family accounted for five.

Inter-species synteny comparison with pomegranate revealed denser and more continuous synteny blocks (14 *TPS* and 27 *MYC* pairs) than with *A*. *thaliana* (three *TPS* and 32 *MYC* pairs), consistent with a closer evolutionary relationship and more conserved genomic architecture between *L. indica* and pomegranate.

Taken together, the intra- and inter-species synteny results suggest that the segmental duplication events have contributed to the expansion of the *LiTPS* and *LiMYC* families in *L. indica*. The Ka/Ks ratios below 1 observed for the duplicated pairs are consistent with purifying selection acting to maintain their functions. The denser synteny with pomegranate than with *Arabidopsis* is consistent with a closer phylogenetic affinity between *L. indica* and pomegranate, adding genomic evidence for understanding the divergence of these gene families.

**Figure 10:**
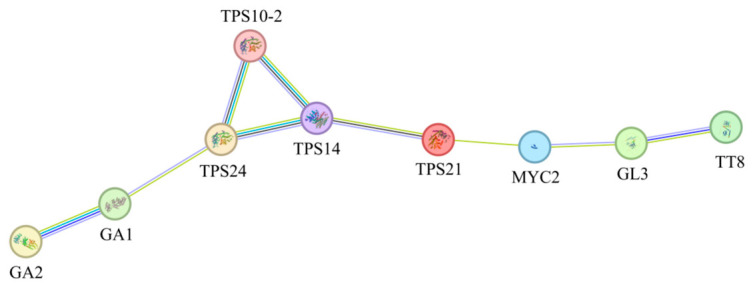
Predicted protein–protein interaction (PPI) network of LiTPS and LiMYC proteins in *L. indica* based on orthologs in *A. thaliana*.

To explore functional associations, we constructed a predicted protein–protein interaction (PPI) network for LiTPS and LiMYC protein based on *A. thaliana* orthologs. This predicted network suggested two main modules: one enriched in terpene synthases and the other containing hormone-related transcription factors. In this predicted network, MYC2 showed a connection with TPS21, suggesting a possible regulatory link. Several TPS proteins, including TPS21, TPS24, TPS14, and TPS10-2, clustered within the terpene synthase module. Whether this clustering reflects functional coordination remains to be experimentally tested, Figure 10.

In the predicted PPI network, the hormone transcription regulation module was centered around *MYC2* as a putative hub. The predicted interactions showed that MYC2 was connected not only to LiTPS21 but also to other bHLH transcription factors (e.g., GL3, TT8) and gibberellin signaling components (GA1, GA2). This predicted connectivity raises the possibility that LiMYC2 may function as a regulatory node that could integrate jasmonate and gibberellin signals, though functional validation is required. If confirmed, such a role would suggest that LiMYC proteins help coordinate terpenoid biosynthesis with broader secondary metabolic response to developmental and environmental cues.

### 2.3. Identification of Two Distinct MeJA-Responsive Gene Modules

**Figure 11:**
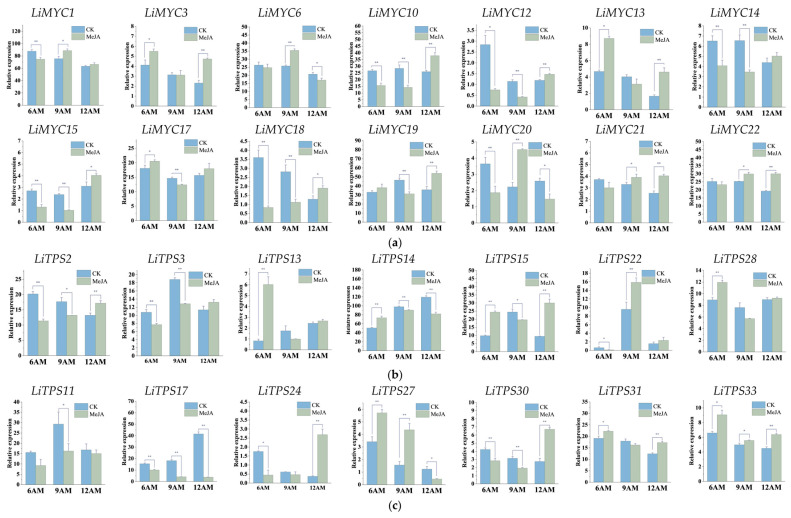
Temporal expression profiles of *LiMYC* transcription factors and *LiTPS* biosynthetic genes in response to 0.25% MeJA treatment. (**a**) Relative expression levels of selected *LiMYC* genes at 6:00, 9:00, and 12:00 under CK and 0.25% MeJA conditions. (**b**) Expression patterns of *LiTPS* genes predicted to be localized in plastids. (**c**) Expression patterns of *LiTPS* genes predicted to be localized in the cytosol. Data represent mean ± SD *n* = 3. Asterisks indicate significant differences between CK and 0.25% MeJA treatments.* *p* < 0.05, ** *p <* 0.01.

qPCR analysis revealed gene-specific rather than uniform activation following MeJA treatment. Among *LiMYC* genes, those phylogenetically grouped with Arabidopsis activators *AtMYC2*–*AtMYC5* (*LiMYC9*, *LiMYC10*, *LiMYC14*) were downregulated after treatment, whereas *LiMYC13* and *LiMYC17*, clustering with repressors *AtJAM1*–*AtJAM5*, showed a transient increase at 06:00 and a subsequent decline at 09:00 (Figure 11). For *LiTPS* genes, *LiTPS2*, *LiTPS3*, and *LiTPS17* (TPS-a) were downregulated, while the chr11-clustered *LiTPS24*, *LiTPS27*, and *LiTPS28* were upregulated at multiple time points. *LiTPS13* (TPS-b) exhibited a 7.33-fold increase at 06:00, possibly associated with predicted JA-responsive cis-elements in its promoter. *LiTPS15* and *LiTPS22* (TPS-g) were also upregulated. These transcriptional patterns are consistent with a role for MeJA in regulating terpenoid biosynthesis, though experimental validation is needed to establish functional relevance.

## 3. Discussion

### 3.1. Floral Scent Characteristics of L. indica ‘Whit III’

Terpenoids constitute the major fraction of floral volatiles in *L indica* [24], and function in defense and plant–insect interactions [25]. We found that the floral scent of *L. indica* ‘Whit III’ was dominated by the monoterpene citronellol and the sesquiterpene *trans*-farnesol. Among 15 other *L. indica* cultivars surveyed previously, *trans*-farnesol was not detected [26], suggesting that *L. indica* ‘Whit III’ has a distinct terpenoid profile. Although its total volatile emission (4.33 μg·g^−1^) is slightly lower than that of the highly fragrant *L. caudata* (5.64 μg·g^−1^) [27,28], the abundance of *trans*-farnesol in this cultivar is noteworthy. *trans*-farnesol has a mild odor and a favorable safety profile [29], which may be of interest for fragrance-related applications, though its sensory contribution is limited by a relatively high odor detection threshold (0.2 mg·kg^−1^) compared with linalool (0.00017 mg·kg^−1^) and geraniol. Gas chromatography–olfactometry (GC–O) would be needed to directly assess the perceptual impact of these volatiles.

### 3.2. Effects of Exogenous MeJA on Volatile Emission: Increased Amount and Advanced Timing

To date, no studies have investigated the response of *Lagerstroemia* to exogenous MeJA [21]. Our results showed that the MeJA treatment both increased the total amount of floral volatiles and advanced the timing of their peak emission in *L. indica* ‘Whit III’.

MeJA significantly stimulated the synthesis of both monoterpenes and sesquiterpenes. This is consistent with reports in *Rosa rugosa* [30], *Chrysanthemum indicum* var. *aromaticum* [31], and various woody species such as *Sitka Spruce* [32] and *Morus notabilis* [33], confirming the conserved role of MeJA in activating secondary metabolism under simulated stress conditions. Notably, species-specific responses have been observed: while many studies highlight linalool accumulation (e.g., *Lavandula angustifolia* [34], *H. coronarium* [20]), our study revealed a strong induction of *trans*-farnesol. Reports of MeJA-induced farnesol synthesis are rare, limited to *Eremochloa ophiuroides* [35] and tea plants [36], where α-farnesol appears de novo. In *L. indica* ‘Whit III’, however, *trans*-farnesol not only accumulates but becomes the dominant volatile. These results suggest that *Lagerstroemia* exhibits a strong sesquiterpene response to MeJA, which may involve species-specific regulatory mechanisms.

MeJA also advanced the timing of emission peak. This temporal shift contrasts with species-specific responses reported in other plants; for example, MeJA delays linalool emission in *S. spruce* [32] but advances it by 6 h in *L. angustifolia* [34]. The earlier emission observed in *L. indica* ‘Whit III’ could represent an adaptive trait. The earlier peak observed here could represent an adaptive trait, potentially allowing the plant to mount a defense or attract natural enemies before significant herbivore pressure occurs [37,38,39], or to synchronize scent emission with pollinator activity [2]. These possibilities remain speculative and would require ecological experiments to be tested.

### 3.3. Candidate MYC and TPS Genes Involved in MeJA-Induced Terpene Synthesis

The JA signaling pathway is known to activate downstream structural genes through transcriptional regulation [34]. According to the canonical model, MeJA promotes the degradation of JAZ repressors, thereby releasing MYC transcription factors to activate target genes [7,9]. Our bioinformatic analyses provided supporting evidence for this mechanism in *Lagerstroemia*:

*Cis*-regulatory elements in the promoter regions were analyzed to assess potential responsiveness to JA signaling. Promoter analysis predicted an enrichment of JA-responsive elements (CGTCA-motif and TGACG-motif) in the *LiTPS* genes compared with *LiMYC* genes. This enrichment may correlate with the strong induction of *LiTPS* genes after the MeJA treatment, but the presence of these motifs alone does not establish regulatory function.

Protein–protein interaction (PPI) analysis based on *Arabidopsis* orthologs identified candidate LiMYC and LiTPS proteins that may form functional associations. The predicted network suggested that LiMYC2 may serve as a central hub, interacting not only with other bHLH factors (e.g., GL3) but also directly with *LiTPS21*. Similar interaction patterns have been reported in other species, such as AtMYC2 binding to *AtTPS21* and *AtTPS11* promoters in *Arabidopsis* [40], ginger lily (HcMYC2 activating *HcTPS*) [20], and osmanthus (OfMYC2-OfMYB21 complex regulating *OfTPS2*) [19].

### 3.4. Expression Patterns and a Potential Negative Feedback Loop

Contrary to the classic model in which MeJA activates positive regulators such as AtMYC2 [41], the *LiMYC* genes phylogenetically related to *AtMYC2*–*AtMYC5* were downregulated after MeJA treatment, whereas *LiMYC13* and *LiMYC17*, which cluster with the JA-signaling repressors *AtJAM1–AtJAM5*, were transiently upregulated at 6:00. This pattern deviates from the simple model in which MeJA universally upregulates positive MYC regulators, and instead suggests the engagement of a negative feedback mechanism. In *Arabidopsis*, JAM proteins suppress JA signaling to maintain metabolic homeostasis. The transient nature of this repressor upregulation in *L. indica* may allow for a rapid but controlled activation of terpenoid biosynthesis. However, alternative explanations should also be considered: the limited sampling time points may have missed the peak activation window of activator-type *LiMYC* genes, which can occur within 1–2 h after JA treatment in *Arabidopsis*; tissue- or developmental-stage-specific regulation may also contribute to the observed patterns; and post-transcriptional control, such as protein stabilization or JAZ-mediated repression, may uncouple transcript levels from actual protein activity.

The expression patterns of *LiTPS* genes also varied across subfamilies and genomic locations. Interestingly, *LiTPS24*, *LiTPS27*, and *LiTPS28*, which are located in a tandem cluster on chromosome 11, were strongly induced by MeJA, whereas other TPS-a family members were downregulated. This suggests that tandem duplication may have facilitated the evolution of JA-inducible sesquiterpene synthases in *L. indica*, possibly through the acquisition or retention of *cis*-regulatory elements in their promoter regions. In support of this, *LiTPS13*, which showed the strongest induction (7.33-fold), contains six JA-responsive *cis*-regulatory elements in its promoter—the highest number among all *LiTPS* genes. This correlation between promoter architecture and expression responsiveness supports the idea that promoter *cis*-element composition is a key determinant of inducibility.

When integrated with our metabolite profiles, these expression patterns suggest a temporally coordinated regulatory program. The early induction (6:00) of *LiTPS13* and *LiTPS27* coincided with the rapid accumulation of monoterpenes and sesquiterpenes observed at 9:00, consistent with a role in initiating terpenoid biosynthesis. Meanwhile, the sustained expression of *LiTPS22* and *LiTPS30* from 9:00 to 12:00 may support prolonged emission during the peak flowering period. Notably, the transient upregulation of the repressor-type *LiMYC13/17* at 6:00 may serve to fine-tune the duration and intensity of this response, preventing excessive metabolic costs. Together, these findings point to a multi-layered regulatory network in which specific LiMYC members control distinct subsets of *LiTPS* genes to achieve precise spatiotemporal control of terpenoid emission in response to MeJA.

### 3.5. Limitations and Perspectives

Several limitations of this study should be acknowledged. First, the experimental design included only three sampling time points, which may not fully capture the dynamics of JA signaling and volatile emission. Second, endogenous JA/JA-Ile levels were not measured; therefore, the effective MeJA dose perceived by the tissues is unknown. Third, the connections between LiMYC transcription factors and *LiTPS* promoters, as well as the enzymatic functions of the identified TPS proteins, remain to be experimentally validated using methods such as yeast one-hybrid, dual-luciferase reporter, and heterologous expression assays.

Our data indicate that the subtle scent of *L. indica* ‘Whit III’ is not due to a lack of biosynthetic capacity, but rather reflects that this capacity is not actively engaged under control group. Exogenous MeJA application enhanced total volatile emission and advanced the peak emission time, suggesting that this cultivar retains the potential for inducible volatile production.

Through genome-wide identification and expression analysis, we cataloged the *LiTPS* and *LiMYC* gene families in *L. indica*. Promoter analysis predicted an enrichment of JA-responsive *cis*-regulatory elements in *LiTPS* genes, and PPI network inferred from *Arabidopsis* orthologs placed LiMYC2 as a putative hub. Together with the observed expression patterns, these data are consistent with a model in which specific *LiMYC* and *LiTPS* genes are temporally coordinated in response to MeJA. Among the identified genes, *LiTPS13* and the tandemly clustered *LiTPS* genes on chromosome 11 were associated with the strongest transcriptional responses, making them candidates for future functional studies.

Future research should aim to experimentally validate the predicted interactions between LiMYC transcription factors and *LiTPS* promoters and to characterize the enzymatic activities of the corresponding LiTPS proteins. In addition, evaluating the ecological effects of MeJA-induced scent changes on pollinator behavior and pest resistance would help clarify the adaptive significance of this response.

## 4. Materials and Methods

### 4.1. Plant Materials and Experimental Treatments

The experiment was conducted in a net house at the Science and Technology Center of Beijing Forestry University. Uniform two-year-old clonal seedlings of *L. indica* ‘Whit III’, exhibiting consistent growth vigor and free from pests or diseases, were selected as test materials. Prior to the experiment, all plants underwent standardized cultivation management to ensure physiological uniformity.

Treatments were applied on a day with stable temperature and humidity. At the bud stage (02:00–04:00), fully opened flowers were removed, retaining only flower buds at a consistent developmental stage. A randomized complete block design was employed. Plants were uniformly sprayed with either 200 mL of 0.25% MeJA in ethanol (treatment group) or 200 mL of 5% ethanol alone (control group, CK).

Samples were collected at three time points post-treatment: 06:00, 09:00, and 12:00 (corresponding to 4, 7, and 10 h after application, respectively). At each time point, six fully opened flowers were randomly selected per plant. Three biological replicates were established for each treatment. Immediately after collection, the samples were placed in sealed headspace vials equipped with biological ice packs and rapidly transported to the laboratory for volatile collection and analysis.

### 4.2. Chemical Reagents

MeJA (CAS: 39924-52-2, GC ≥ 98%), the internal standard 3-octanol (CAS: 589-98-0, GC ≥ 98%), and anhydrous ethanol were purchased from Shanghai Yuanye Bio-Technology Co., Ltd. (Shanghai, China). HPLC-grade n-hexane was used for standard dilution.

### 4.3. Volatile Collection and SPME-GC-MS Analysis

Fresh whole flowers (0.2 g, approximately two flowers) were accurately weighed and placed into 20 mL headspace vials, gently compacted to prevent damage to the extraction fiber. One microliter of 3-octanol internal standard solution (0.82 μg·μL^−1^) was added to each vial, which was then immediately sealed. The samples were stored at 4 °C until analysis.

Prior to use, a Divinylbenzene/Carboxen/Polydimethylsiloxene (DVB/CAR/PDMS) SPME fiber (50/30 μm) was conditioned at 250 °C for 30 min. For extraction, vials were equilibrated in a 45 °C water bath for 10 min. The SPME fiber was then inserted into the vial headspace and exposed at 45 °C for 50 min. Following adsorption, the fiber was transferred to the GC injection port for thermal desorption at 200 °C for 5 min in splitless mode.

Volatile compounds were separated and detected using a Shimadzu GCMS-QP2010 (Shimadzu Corporation, Kyoto, Japan). Plus system equipped with a DB-5MS capillary column (30 m × 0.25 mm × 0.25 μm; Agilent Technologies, Santa Clara, CA, USA). Helium (99.99%) served as the carrier gas at a constant flow rate of 1.375 mL·min^−1^. The injector temperature was maintained at 200 °C. The oven temperature program was as follows: initial hold at 40 °C for 2 min, ramped to 200 °C at 5 °C·min^−1^, and held for 6 min. Mass spectrometry conditions included an interface temperature of 250 °C, an ion source temperature of 200 °C, electron impact (EI) ionization at 70 eV, and a scan range of m/z 30–500.

Identification and Quantification: Compounds were identified by matching mass spectra against the NIST 27 and Wiley 139 libraries (match quality > 80%), corroborated by retention indices (RI) and literature data. Quantification was performed using 3-octanol as an internal standard. The content of each aroma component (μg·g^−1^) was calculated using the formula:

Content (μg·g^−1^) = (*A_compound_*/*A_IS_*) × *C_IS_* × *V_IS_* × (1/*W_sample_*)

where *A_compound_* and *A_IS_* are the peak areas of the compound and internal standard, respectively; *C_IS_* is the concentration of the internal standard (0.82 μg·μL^−1^); *V_IS_* is the volume added (1 μL); and *W_sample_* is the fresh weight of the sample (g).

### 4.4. Statistical Analysis

All data are presented as means ± standard deviation (SD) of three biological replicates. One-way analysis of variance (ANOVA) was performed using SPSS 21.0 (IBM Corp., Armonk, NY, USA). Significant differences between treatments were determined using Duncan’s multiple range test (*p* < 0.05). Graphs were generated using Origin 8.0 (OriginLab Corp., Northampton, MA, USA). The same significance analysis methods and plotting procedures were applied to both the volatile profiling data and the qPCR results.

### 4.5. Genome-Wide Identification of LiTPS and LiMYC Gene Families

Members of the *TPS* and *MYC* gene families in *L. indica* were identified using a combined homology-based and profile-hidden Markov model (HMM) approach. First, protein sequences of *A. thaliana AtTPS* and *AtMYC* families were retrieved from the TAIR database (https://www.arabidopsis.org/) and used as queries for BLASTp searches (BLAST+ v2.9.0) [42] against the L. indica proteome. Second, HMM profiles for *TPS* (PF01397, PF03936) and *MYC* (PF00010, PF14215) domains were downloaded from the Pfam database and used to search the *L. indica* proteome using HMMER v3.0, with an E-value threshold of <10^−3^. Candidate sequences obtained from both methods were intersected and further validated for domain integrity using the SMART database (https://smart.embl.de/). Only proteins containing complete target domains were retained. Chromosomal localization and systematic naming were performed using TBtools v2.442 [43]. The ExPASy database (https://web.expasy.org/protparam/, accessed on 5 March 2026) was used to compute the molecular weight (Mw), isoelectric points (pI), grand average of hydrophobicity (GRAVY), and subcellular localization.

### 4.6. Phylogenetic Tree Construction

To elucidate evolutionary relationships, full proteomes of tomato (*Solanum lycopersicum*) and pomegranate (*Punica granatum*) were downloaded from EnsemblPlants (https://plants.ensembl.org/). *TPS* and *MYC* members in these species were identified using the same criteria described above. Multiple sequence alignments of TPS and MYC proteins from *L. indica*, *A. thaliana*, *S. lycopersicum*, and *D. officinale* were performed. Phylogenetic trees were constructed using the Neighbor-Joining (NJ) method in MEGA v12.0.15 [44], with 1000 bootstrap replications; all other parameters were set to default. The resulting trees were visualized and annotated using the Evolview online tool (https://evolgenius.info/, accessed on 5 March 2026).

### 4.7. Conserved Motif and Gene Structure Analysis

Conserved motifs in LiTPS and LiMYC proteins were identified using the MEME suite (https://meme-suite.org/, accessed on 5 March 2026), with parameters set to identify up to 10 motifs (maximum width: 102 amino acids). Gene structure information (exon–intron organization) was extracted from the *L. indica* genome annotation file. Both motif distribution and gene structure maps were generated and integrated using TBtools v2.442 [43] and the GSDS 2.0 server (https://gsds.gao-lab.org/, accessed on 5 March 2026).

### 4.8. Promoter Cis-Regulatory Element Analysis

Promoter sequences (2000 bp upstream of the translation start site) for all *LiTPS* and *LiMYC* genes were extracted from the *L. indica* genome using TBtools v2.442 [43]. These sequences were submitted to the PlantCARE database (https://bioinformatics.psb.ugent.be/webtools/plantcare/html/, accessed on 5 March 2026) for the prediction and functional annotation of cis-acting elements. The distribution of these elements was visualized using TBtools.

### 4.9. Synteny Analysis

Intra-genomic (within *L. indica*) and inter-genomic (between *L. indica* vs. *A. thaliana* and *L. indica* vs. *P. granatum*) synteny analyses for *TPS* and *MYC* families were performed using MCScanX v1.0.0 based on whole-genome data and annotation files. Synteny plots were visualized using TBtools v2.442 [43]. Additionally, the ratio of non-synonymous to synonymous substitution rates (Ka/Ks) for syntenic gene pairs was calculated using TBtools to evaluate evolutionary selection pressures.

### 4.10. Protein–Protein Interaction (PPI) Network Prediction

To predict functional interactions, protein sequences of *LiTPS* and *LiMYC* families were used as queries against the *A. thaliana* reference proteome in the STRING database (https://cn.string-db.org/). High-confidence interaction networks were constructed based on orthologous relationships. The resulting PPI networks were visualized using the built-in tools of the STRING platform to infer potential regulatory modules and interaction patterns in *L. indica*.

### 4.11. Quantitative Real-Time PCR (qRT-PCR) Analysis of LiMYC and LiTPS Genes

Total RNA was extracted from pooled whole flower samples (approximately 0.1 g per replicate, three biological replicates pooled) using an RNAprep Pure Plant Kit (Tiangen, Beijing, China), with one extraction per sample. First-strand cDNA was synthesized using HiScript III 1st Strand cDNA Synthesis Kit (+gDNA wiper) (Vazyme, Nanjing, China). qRT-PCR was performed using a LightCycler 480II (Roche, Basel, Switzerland) with the AceQ qPCR SYBR Green Master Mix (Vazyme, Nanjing, China). The 2^−ΔΔCt^ technique was used to examine the expression data from three biological replicates [45]. We selected EF-1α as the internal reference gene [46].

## Data Availability

The original contributions presented in this study are included in the article/Supplementary Material. Further inquiries can be directed to the corresponding author.

## Supplementary Materials

The following supporting information can be downloaded at: https://www.mdpi.com/article/10.3390/plants15111600/s1, Figure S1: Localization of TATA-box and selected JA-related *cis*-regulatory elements in the promoter regions; Table S1: Detailed results of volatile compound detection; Table S2: Physicochemical properties and subcellular localization predictions of LiTPS proteins; Table S3: Gene expression results from qPCR analysis.
