# Supplementary figures and images for: Identification of LiMYC and LiTPS Gene Families Involved in MeJA-Induced Terpene Accumulation in Lagerstroemia indica ‘Whit III’
Source: Plants (Basel). 2026 May 22;15(11):1600. doi: 10.3390/plants15111600 (PMC13258938; doi:10.3390/plants15111600)

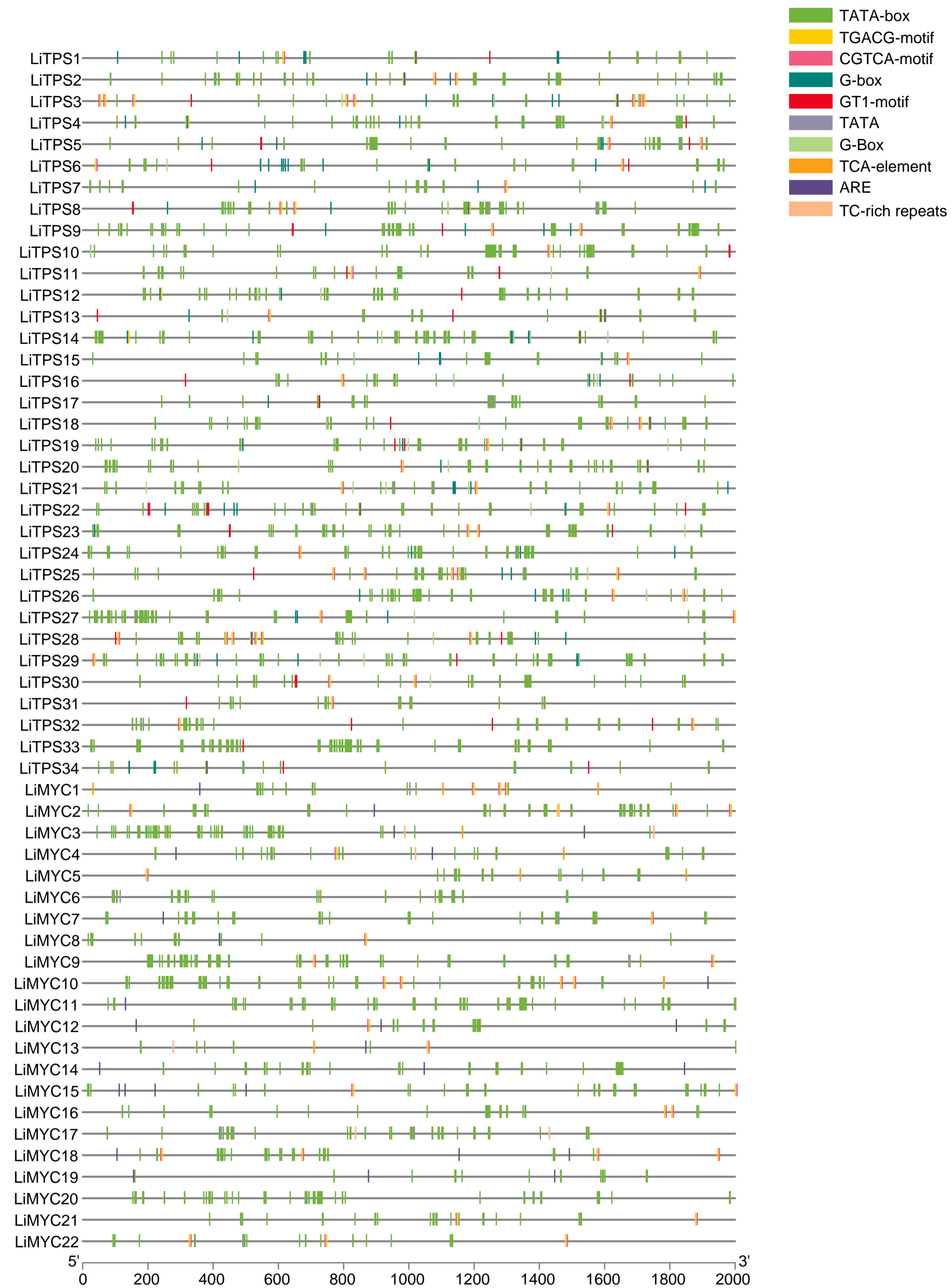

Supplement: Supplementary file 1 [file plants-15-01600-s001.zip › Figure S1 Localization of TATA-box and selected JA related cis regulatory elements in the promoter regions.pdf]
